# Opposing Functions of Akt Isoforms in Lung Tumor Initiation and Progression

**DOI:** 10.1371/journal.pone.0094595

**Published:** 2014-04-10

**Authors:** Nicolle M. Linnerth-Petrik, Lisa A. Santry, James J. Petrik, Sarah K. Wootton

**Affiliations:** 1 Department of Pathobiology, Ontario Veterinary College, University of Guelph, Guelph, Ontario, Canada; 2 Department of Biomedical Science, Ontario Veterinary College, University of Guelph, Guelph, Ontario, Canada; McMaster University, Canada

## Abstract

**Background:**

The phosphatidylinositol 3-kinase–regulated protein kinase, Akt, plays an important role in the initiation and progression of human cancer. Mammalian cells express three Akt isoforms (Akt1–3), which are encoded by distinct genes. Despite sharing a high degree of amino acid identity, phenotypes observed in knockout mice suggest that Akt isoforms are not functionally redundant. The relative contributions of the different Akt isoforms to oncogenesis, and the effect of their deficiencies on tumor development, are not well understood.

**Methods:**

Here we demonstrate that Akt isoforms have non-overlapping and sometimes opposing functions in tumor initiation and progression using a viral oncogene-induced mouse model of lung cancer and Akt isoform-specific knockout mice.

**Results:**

Akt1 ablation significantly delays initiation of lung tumor growth, whereas Akt2 deficiency dramatically accelerates tumorigenesis in this mouse model. Ablation of Akt3 had a small, not statistically significant, stimulatory effect on tumor induction and growth by the viral oncogene. Terminal deoxynucleotidyl transferase–mediated dUTP nick end labeling and Ki67 immunostaining of lung tissue sections revealed that the delayed tumor induction in Akt1^−/−^ mice was due to the inhibitory effects of Akt1 ablation on cell growth and survival. Conversely, the accelerated growth rate of lung tumors in Akt2^−/−^ and Akt3^−/−^ mice was due to increased cell proliferation and reduced tumor cell apoptosis. Investigation of Akt signaling in tumors from Akt knockout mice revealed that the lack of Akt1 interrupted the propagation of signaling in tumors to the critical downstream targets, GSK-3α/β and mTOR.

**Conclusions:**

These results demonstrate that the degree of functional redundancy between Akt isoforms in the context of lung tumor initiation is minimal. Given that this mouse model exhibits considerable similarities to human lung cancer, these findings have important implications for the design and use of Akt inhibitors for the treatment of lung cancer.

## Introduction

Lung cancer accounts for 27% of all cancer-related deaths each year making it the most common cause of cancer-related death in both males and females. With a dismal 5-year survival rate of only 16%, there is an urgent need to better understand the etiology and carcinogenic mechanisms involved in the development of lung cancer in order to identify optimal therapeutic strategies.

The study of oncogenic retroviruses has been instrumental in informing our understanding of oncogenes and the molecular basis of cancer. Jaagsiekte sheep retrovirus (JSRV) is an acutely oncogenic betaretrovirus that causes adenocarcinomas in the distal airways of sheep through the activation of the PI3K/Akt and MAPK signaling pathways [1]. The ability of JSRV to cause lung tumors in sheep that are histologically and phenotypically similar to those frequently found in humans, particularly that of never smokers, make it an attractive model for understanding the etiology and carcinogenesis of human lung cancer [2]–[4]. Unlike most replication-competent retroviruses, which cause cancer by insertional activation of cellular oncogenes or by acquisition of cellular oncogenes, the envelope protein of JSRV is itself a potent oncogene that when expressed in mouse [5] or sheep lungs [6] is sufficient to induce lung cancer. We have developed a tractable mouse model to study lung cancer induced by the JSRV envelope protein (Jenv) that involves delivering the Jenv gene to the mouse respiratory tract using a replication defective adeno-associated virus (AAV) vector. Tumors induced in mice resemble those of non-small-cell lung carcinoma (NSCLC) in never smokers, both histologically and with respect to activated signal transduction pathways [7]. Notably, the PI3K/Akt pathway is among the most significant pathways activated by Jenv.

A common feature of many human cancers, including lung cancer [8], [9], is the unregulated activation of the Akt pathway. The protein kinase, Akt, is a major signal transducer of the phosphatidylinositol 3-kinase (PI3K) pathway and plays a pivotal role in the maintenance of many cellular processes including cell growth, proliferation, survival and metabolism [10]. In mammals, three distinct genes encode for Akt1 (PKBα), Akt2 (PKBβ) and Akt3 (PKBγ) and the encoded proteins share ∼80% amino acid sequence identity [11]. Phenotypes observed in knockout mice suggest that Akt isoforms are not functionally redundant. Akt1^−/−^ mice display impaired overall growth [12], Akt2^−/−^ mice are unable to maintain glucose homeostasis [13], and Akt3^−/−^ mice have a reduction in brain size [14]. Whereas Akt1 and Akt2 are ubiquitously expressed, Akt3 displays a more restricted tissue distribution and is highly expressed in the testes and brain [15]. Until recently, it was believed that all three isoforms functioned to increase tumor cell survival and proliferation making Akt an attractive therapeutic target [16]. Recent *in vivo* studies, however, have demonstrated isoform-specific functions in tumorigenesis [17]–[23] and suggest that Akt isoforms are responsible for distinct biological outcomes.

In this study, we tested the hypothesis that Akt isoform ablation will have distinct effects on lung tumor initiation and progression in a viral oncogene-induced mouse model of lung cancer that resembles lung cancer in never-smokers [7]. Our results demonstrate that Akt1 ablation suppresses lung tumor initiation due to inhibitory effects on cell proliferation and survival. Akt2 ablation on the other hand, dramatically accelerates lung tumor initiation via enhanced proliferation and suppression of apoptosis suggesting that Akt2 activity may have a protective effect in lung cancer. Akt3 ablation moderately accelerated lung tumor progression and extralobular metastasis. Therefore, the degree of functional redundancy between the Akt isoforms in the context of lung tumorigenesis is minimal and should be considered in the development of Akt-targeted therapies.

## Results

### All three Akt isoforms are expressed in the mouse lung

**Figure 1:**
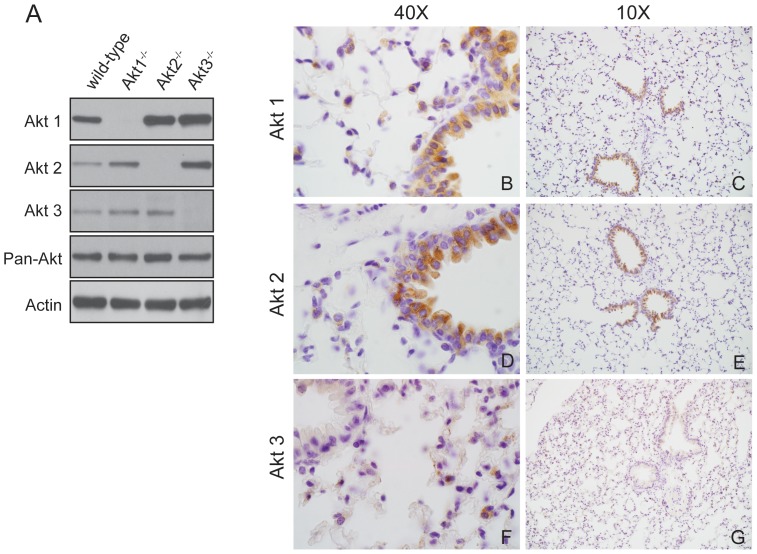
All three Akt isoforms are expressed in mouse lungs. (A) Representative western blot analysis of 80 ug of total protein extracted from the lungs of 8-week-old C57BL/6 mice probed with a panel of Akt isoform-specific antibodies (Akt1, Akt2 and Akt3) as well as a pan-Akt antibody. An anti-actin antibody was used to demonstrate equal loading. Immunohistochemical staining of 8-week-old C57BL/6 mouse lungs with Akt1 (B and C), Akt2 (D and E) and Akt3 (F and G) specific antibodies.

To determine the relative expression level of Akt isoforms within mouse lungs, total cell lysates were prepared from the lung tissue of 8-week-old wild type (WT), Akt1^−/−^, Akt2^−/−^, and Akt3^−/−^ C57BL/6 mice and probed with antibodies specific for Akt1, Akt2, Akt3, and pan-Akt. The results demonstrated that all three Akt isoforms are expressed at detectable levels in WT lungs and that lungs of Akt1^−/−^, Akt2^−/−^, and Akt3^−/−^ mice do not express Akt1, Akt2, or Akt3, respectively (Figure 1A). In terms of compensatory expression, it appears that Akt1^−/−^ and Akt3^−/−^ mice express slightly elevated levels of Akt2 whereas Akt2^−/−^ and Akt3^−/−^ mice express slightly elevated levels of Akt1 (Figure 1A). To determine which cell types in the lung express individual Akt isoforms, lung sections from an 8-week-old WT mouse were stained for Akt1, Akt2, and Akt3. As shown in Figure 1B, Akt1 and Akt2 are expressed in alveolar type II pneumocytes (ATII) as well as Clara cells lining the terminal bronchioles, whereas Akt3 is primarily expressed in ATII cells. Note that Akt1 is more highly expressed in ATII cells (Figure 1B), which are the putative tumor originating cells, whereas Akt1 and Akt2 are expressed at near equivalent levels in Clara cells, which are not susceptible to transformation [5]. These results indicate that all three Akt isoforms are expressed at detectable levels in the lung but that the cellular distribution of Akt isoform expression differs among all three isoforms.

### Akt1 ablation delays, whereas Akt2 ablation accelerates the development of lung tumors

**Figure 2:**
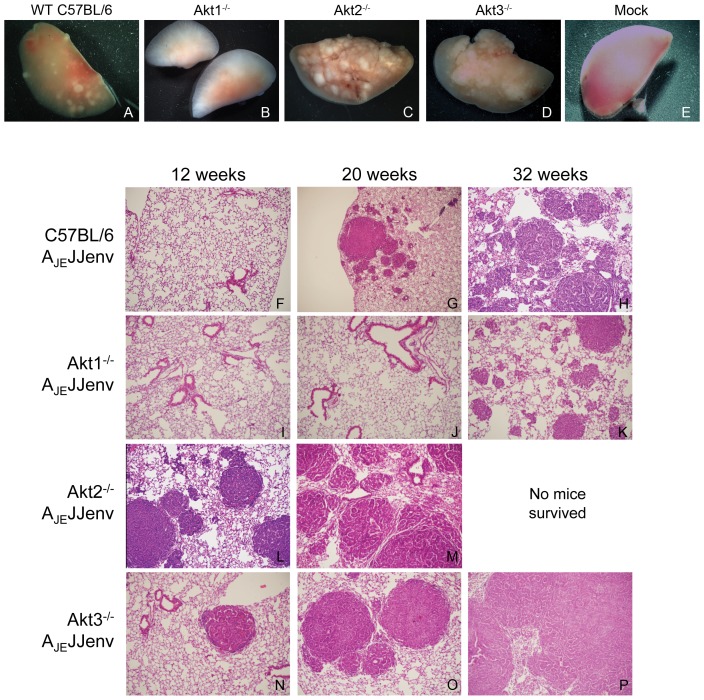
Akt 1 is required for initiation and progression of lung tumors whereas Akt2 appears to be protective against tumorigenesis. Representative macroscopic images of individual lung lobes harvested from A_JE_JJenv infected WT (A), Akt1^−/−^ (B), Akt2^−/−^ (C), and Akt3^−/−^ (D) mice as well as mock infected mice (E) at 20 weeks post-infection. Representative images of hematoxylin and eosin stained sections from the lungs of WT (F to H), Akt1^−/−^ (I to K), Akt2^−/−^ (L and M) and Akt3^−/−^ (N to P) mice infected with A_JE_JJenv and harvested at 12, 20 and 32 weeks post-infection (4× magnification). Note that none of the Akt2^−/−^ infected mice survived past 20 weeks post-infection.

To investigate the role of individual Akt isoforms in lung tumorigenesis, 7-week-old WT, Akt1^−/−^, Akt2^−/−^ and Akt3^−/−^ mice were infected with 1×10^11^ vector genomes (vg) of a replication defective AAV vector expressing the potently oncogenic envelope protein from JSRV (A_JE_JJenv). Note that transduction efficiency of AAV vectors is not influenced by the Akt isoform status of the mice (Figure S1). Mice were euthanized at 12 (early neoplastic lesions), 20 (established tumors) and 32 (advanced neoplasms) weeks post-infection (minimum of 5 mice/group) and lung tissue collected for histological analysis. H&E staining of lung sections at the three defined time points revealed that Akt1 plays a critical role in lung tumor initiation in this model since ablation of Akt1 resulted in a significant reduction in tumors as well as a delay in the onset of tumorigenesis (Figure 2B, I–K). In fact, only 1 out 5 mice had tumors by 32 weeks post-infection (Figure 2K). Conversely, Akt2 appears to be protective against viral oncogene-induced lung tumorigenesis. Akt2^−/−^ mice infected with A_JE_JJenv developed a substantial lung tumor burden (Figure 2L and M) in a significantly shorter period of time relative to their WT counterparts (Figure 2F and G) and most did not survive past the 20-week time point (Figure 2C). Even at 12 weeks post infection, 5 out of 5 Akt2^−/−^ mice had accelerated lung tumorigenesis (Figure 2L). All Akt3^−/−^ mice exhibited multiple focal lesions at 12 and 20 (Figure 2D) weeks post-infection and by 32 weeks mice showed signs of respiratory distress due to excessive tumor burden (Figure 2N-P). Note that 2 out of 5 Akt3^−/−^ mice had to be euthanized prior to the 32-week time point due to respiratory distress. Taken together, these results suggest that Akt1 is essential, whereas Akt2 and to some extent Akt3 are protective against viral oncogene-induced lung tumorigenesis. Additionally, this dependence on Akt1 highlights the importance of the PI3K/Akt pathway over other pathways such as the MEK/ERK pathway in Jenv-induced lung tumor initiation.

**Figure 3:**
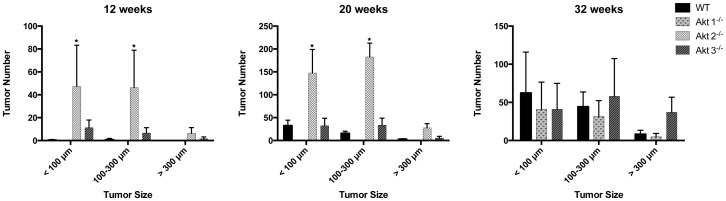
Ablation of Akt2 dramatically enhances the initiation of lung tumorigenesis in A_JE_JJenv infected mice. WT, Akt1^−/−^, Akt2^−/−^ and Akt3^−/−^ mice infected with A_JE_JJenv were euthanized at 12 (A), 20 (B) and 32 (C) weeks post infection and the number of lung tumors less than 100 μm, between 100 and 300 μm, and greater than 300 μm were quantified. Note that since all Akt2^−/−^ mice died around the 20-week time point there is no data for Akt2^−/−^ mice at the 32-week time point. Three lung lobes from three randomly selected mice per group were sectioned until the maximum surface area was exposed at which point all tumors within each lung lobe were counted. Bars on the graph labeled with an asterisk are statistically different (p<0.05).

To quantify tumor burden, three mice from each group were randomly selected and three randomly selected lung lobes from each mouse were sectioned and H&E stained. Total number of tumors in each of three lung lobes were counted and assigned to one of three categories: <100 μm (small), 100–300 μm (medium) or >300 μm (large). At 12 weeks post-infection with A_JE_JJenv, the Akt2^−/−^ mice had a substantial and statistically significant greater number of small and medium sized lung tumors as compared to Akt3^−/−^ mice, which had only a few detectable tumors, and WT and Akt1^−/−^ mice, which had no detectable tumors (Figure 3A). By 20 weeks post-infection, small and medium sized tumors were visible in both the WT and Akt3^−/−^ mice, but the Akt2^−/−^ mice had five times as many small and medium sized lung tumors (Figure 3B), suggesting that tumors were initiating and proliferating at a significantly faster rate in the Akt2^−/−^ mice. Note that all of the A_JE_JJenv infected Akt2^−/−^ mice had to be euthanized between 17 and 20 weeks post-infection due to excessive lung tumor burden. By 32 weeks post-infection, small and medium sized tumors were detectable in only one of five Akt1^−/−^ mice and while Akt3^−/−^ mice had greater numbers of large tumors (>300 μm) compared to WT and Akt1^−/−^ mice (Figure 3C), this was not statistically significant. This analysis revealed that, at both early and late time points, A_JE_JJenv infected Akt2^−/−^ mice harbored significantly greater numbers of hyperplastic foci/tumors than WT, Akt1^−/−^ and Akt3^−/−^ mice, suggesting that ablation of Akt2 enhances tumor initiation.

### Lung tumors induced in Akt1^−/−^, Akt2^−/−^, Akt3^−/−^ and WT mice are SPC positive and CC10 negative

**Figure 4:**
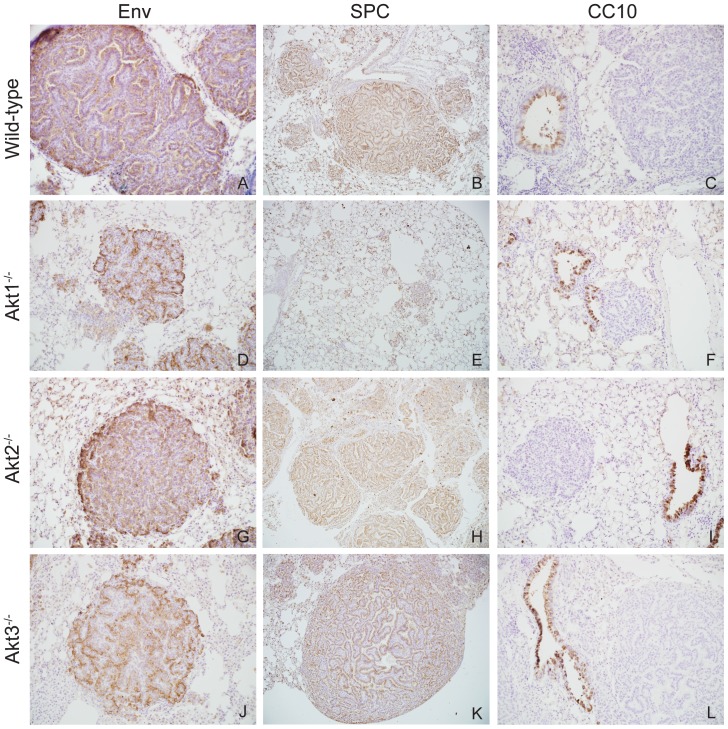
Lung tumors uniformly express Jenv and SPC irrespective of Akt isoform status. Immunostaining for Jenv, SPC, and CC10 expression in lung tissue from A_JE_JJenv infected WT (A to C), Akt1^−/−^ (D to F), Akt2^−/−^ (G to I), and Akt3^−/−^ (J to L) mice at 10× magnification. Representative images of advanced neoplastic lesions show robust Jenv expression within all cells of the lung tumor (A, D, G, J). Representative images show lung tumors staining uniformly positive for SPC (B, E, H, K) and negative for CC10 (C, F, I, L).

Immunohistochemical staining of lung tissue sections from A_JE_JJenv infected WT, Akt1^−/−^, Akt2^−/−^, and Akt3^−/−^ mice with a Jenv-specific monoclonal antibody revealed that all tumors uniformly express the viral oncogene suggesting that tumor initiation in this model was dependent on Jenv expression (Figure 4A, D, G and J). To investigate whether Akt isoform ablation had an effect on the cellular composition of the tumor, lung sections were stained with a surfactant protein C (SPC) specific antibody as a marker for ATII cells and a Clara cell secretory protein (CC10) specific antibody as a marker for non-ciliated secretory epithelial cells lining the primary bronchioles of the lung. As with the WT mice, all lung tumors that formed in the Akt knockout mice were SPC positive (Figure 4B, E, H and K) and CC10 negative (Figure 4C, F, I and L) indicating that the tumors were of ATII cell origin. All staining with isotype control antibodies was negative (data not shown).

### Akt1 ablation inhibits cell proliferation and promotes apoptosis of neoplastic cells

**Figure 5:**
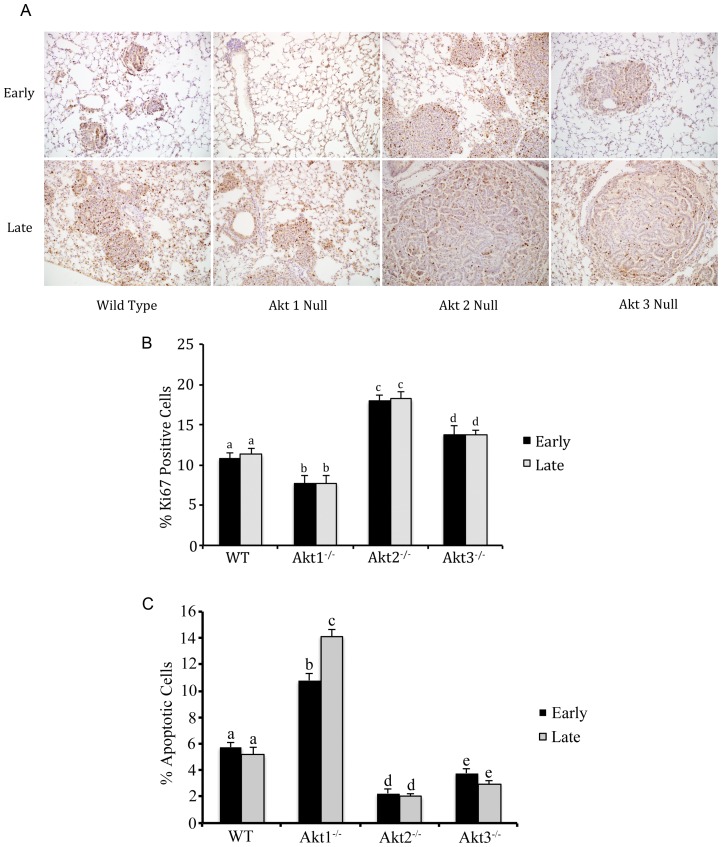
Akt1 ablation inhibits cell proliferation and promotes apoptosis of lung epithelia in A_JE_JJenv infected mice. (A) Lung tissue sections from WT, Akt1^−/−^ and Akt3^−/−^ mice at 12 (early) and 32 (late) weeks post-infection and Akt2^−/−^ mice at 12 (early) and 20 (late) weeks post-infection stained with an antibody against the proliferation marker Ki67. (B) Graphical representation of the number of Ki67 positive cells. The number of Ki67-positive (proliferating) cells was measured in sections of lung derived from three randomly selected mice of each genotype and three randomly selected fields per mouse. The bars show the mean percentage of proliferating cells ± SE of the mean for each genotype. (C) Quantification of TUNEL-positive apoptotic cells in early and late (advanced) neoplastic lesions from WT, Akt1^−/−^, Akt2^−/−^ and Akt3^−/−^ A_JE_JJenv infected mice. The number of TUNEL-positive cells was measured in sections of lung derived from three randomly selected mice of each genotype and three randomly selected fields per mouse. The bars show the mean percentage ± the SE of the mean of TUNEL-positive cells in early and late lesions. For figures B and C, 2-way ANOVA and Bonferonni's correction was used and bars on the graph with different letters are statistically different (p<0.05).

To determine whether Akt1 ablation inhibits cell proliferation, lung tissue sections from A_JE_JJenv infected Akt1^−/−^ mice at 12 (representing early neoplastic lesions) and 32 (representing advanced neoplasms) weeks post-infection were stained for the Ki67 proliferation marker and compared with similarly stained sections from WT, Akt2^−/−^ and Akt3^−/−^ infected mice (Figure 5A). This comparison revealed that the ablation of Akt1 significantly inhibited cell proliferation while the ablation of Akt2 and Akt3 enhanced cell proliferation (Figure 5B). These results also show that proliferation rates are comparable between early and late lesions irrespective of genotype, suggesting that tumor cells in advanced lesions do not adapt to the loss of Akt isoforms.

To address the role of Akt isoforms in cell survival in early and advanced neoplastic lesions, sections of lung from three randomly selected mice per group at early and late time points post-infection were analyzed for apoptosis using the TUNEL assay. This analysis identified a greater than 2-fold increase in the number of apoptotic cells in early neoplastic lesions and a 3-fold increase in the number of apoptotic cells in advanced neoplasms from Akt1^−/−^ infected mice relative to WT mice (Figure 5C). Conversely, ablation of Akt2 resulted in a nearly 3-fold reduction in the number of apoptotic cells in both early and advanced lesions relative to WT mice (Figure 5C). A reduction in the number of apoptotic cells was also observed in the Akt3^−/−^ mice, but while this reduction was significant, it was not as dramatic as was observed in the Akt2^−/−^ mice (Figure 5C). Taken together, these observations suggest that Akt1 deficiency may impede the growth of preneoplastic lesions and established tumors by inhibiting both cell proliferation and cell survival.

### Immunoblot analysis of Akt isoform expression and Akt pathway activation

**Figure 6:**
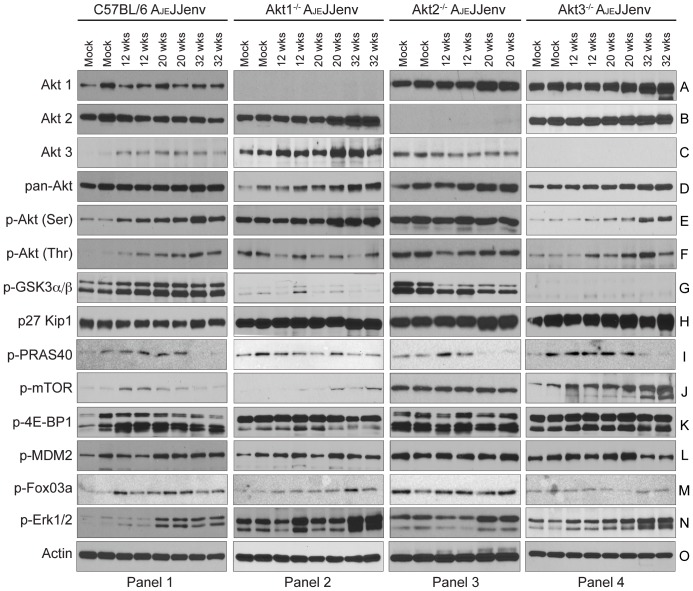
Akt isoform expression and Akt pathway activation in lungs from WT, Akt1^−/−^, Akt2^−/−^ and Akt3^−/−^ mice infected with A_JE_JJenv. Representative western blot images of 80_JE_JJenv infected WT (panel 1), Akt1^−/−^ (panel 2), Akt2^−/−^ (panel 3), and Akt3^−/−^ (panel 4) mice at 12, 20 and 32 weeks post-infection probed with Akt isoform specific antibodies (A to C), pan-Akt (D), a panel of phospho-specific (p) antibodies (E to N) and actin (O) as a loading control.

Total cell lysates prepared from mouse lungs at 12, 20 and 32 weeks post-infection with A_JE_JJenv were probed by western blot with antibodies against Akt1, Akt2 and Akt3 to determine whether Akt isoform expression levels varied relative to normal lung. In WT mice, the total level of Akt1 expression did not differ considerably from that of mock infected mice (Figure 6A, panel 1) whereas the amount of Akt2 expression declined as tumors progressed (Figure 6B, panel 1). Akt3 levels were increased in the infected WT mice but did not continue to increase over time (Figure 6C, panel 1). In the case of the Akt1^−/−^ mice, both Akt2 and Akt3 expression levels increased over time (Figure 6B and C, panel 2), as did the total amount of Akt (Figure 6D, panel 2). In Akt2^−/−^ mice at 20 weeks post-infection, the amount of Akt1 expression was increased and this corresponded to an increase in total Akt (Figure 6A and D, panel 3). Similarly, in the Akt3^−/−^ mice the amount of Akt1 expression increased over time and this corresponded to an increase in total Akt (Figure 6A and D, panel 4). Therefore, there appears to be some compensatory increase in expression of the remaining Akt isoforms in isoform-ablated mice, most notably in Akt1^−/−^ mice.

Evaluation of the amount of activated Akt as determined by phosphorylation of both the serine (Ser473) and threonine (Thr308) residues revealed that phosphorylation gradually increased in the WT, Akt1^−/−^ and Akt3^−/−^ mice over time (Figure 6E and F, panels 1, 2 and 4) whereas the amount of activated Akt in the Akt2^−/−^ mice did not change over time (Figure 6E and F, panel 3), despite the fact that tumor growth in these mice was very aggressive (Figure 2G and H). One explanation for the lack of increased phosphorylation of Akt in the Akt2^−/−^ mice could be that the basal level of activated Akt was already relatively high in these mice.

To determine whether Akt isoform ablation disrupted activation of the Akt pathway, downstream targets of Akt were assessed in A_JE_JJenv infected WT, Akt1^−/−^, Akt2^−/−^ and Akt3^−/−^ mice. As was reported previously, in WT mice there was a concomitant increase over time in the amount of phosphorylated pGSK-3α/β relative to mock-infected mice (Figure 6G, panel 1). Interestingly, neither Akt1^−/−^ nor Akt3^−/−^ mice had detectable levels of phosphorylated pGSK-3α/β (Figure 6G, panels 2 and 4, respectively) whereas Akt2^−/−^ mice showed a decrease in pGSK-3α/β phosphorylation as tumor burden increased (Figure 6G, panel 3). P27 Kip1 levels remained relatively constant irrespective of mouse strain or tumor burden (Figure 6H). The predominant mechanism by which Akt promotes cell growth is through phosphorylation and inactivation of either TSC2 [24], [25] or PRAS40 [26], [27]. Despite numerous attempts, we were unable to detect phosphorylated TSC2 in our tissue samples (data not shown). PRAS40 phosphorylation increased in conjunction with tumor burden in WT, Akt2^−/−^ and Akt3^−/−^ mice, however, at the latest time point post-infection, when tumor burden was substantial, PRAS40 phosphorylation was inhibited (Figure 6I, panel 1, 3 and 4). This was not the case for Akt1^−/−^ mice as PRAS40 phosphorylation was detectable at all time points (Figure 6I, panel 2). Phospho-mTOR levels increased in conjunction with increased tumor burden in Akt1^−/−^ and Akt3^−/−^ mice (Figure 6J, panels 2 and 4), remained constant in Akt2^−/−^ mice (Figure 6J, panel 3) and tapered off in WT mice with late stage tumors (Figure 6J, panel 1). Phospho-4E-BP1, which is a downstream target of mTOR, followed the same pattern of activation as phospho-mTOR in the WT mice where in late stage tumors it tapered off (Figure 6K, panel 1). However, phospho-4E-BP1 levels remained constant in all three knockout mice irrespective of tumor burden (Figure 6K, panels 2–4). Phospho-MDM2, which when phosphorylated by Akt enhances cell survival, was slightly increased in WT mice (Figure 6L, panel 1), but remained relatively unchanged in the Akt knockout mice with the exception of a slight decrease in Akt3^−/−^ mice in advanced neoplasms (Figure 6K, panel 4). Activated Akt can phosphorylate FoxO3a and BAD, leading to their inactivation and promotion of cell survival [28]–[30]. We observed an increase in FoxO3a phosphorylation in WT and Akt1^−/−^ mice concomitant with tumor burden (Figure 6M, panels 1 and 2), but FoxO3a phosphorylation remained constant in Akt2 and Akt3 mice irrespective of tumor burden (Figure 6M, panels 3 and 4). Despite many attempts, we were unable to detect phosphorylated BAD (data not shown). Lastly, as expected, ablation of Akt isoforms had no effect on Erk1/2 activation as phospho-Erk1/2 increased over time in all three knockout mice as well as in the WT mice (Figure 6N). In summary, Akt isoform ablation predominantly influences GSK-3α/β and mTOR signaling in this model.

## Discussion

In view of the fact that Akt modulates a multitude of cellular processes including cell proliferation, survival, metabolism and metastasis, all of which are hallmarks of cancer [31], it is not surprising that Akt is one of the most frequently hyperactivated kinases in human cancers [32], including lung cancer [33]–[35]. However, it is not completely understood how individual Akt isoforms function in the context of tumorigenesis. The experiments described in this report addressed the biological specificity of individual Akt isoforms in the context of a viral oncogene-induced mouse model of lung cancer. We report that Akt1 ablation inhibits the incidence and development of lung tumors, Akt2 deficiency markedly accelerates lung tumor initiation and Akt3 ablation slightly enhances tumor progression in this mouse model of lung cancer.

The histopathology between Jenv-induced tumors arising in WT, Akt1^−/−^, Akt2^−/−^, and Akt3^−/−^ mice was similar suggesting that ablation of different isoforms does not result in the development of biologically distinct neoplasms. Other than in the Akt3 knockout mice, where 4 out of 20 mice exhibited extralobular tumor growth, the ablation of individual Akt isoforms did not appear to have an effect on local invasiveness or metastatic potential of Jenv-induced lung tumors. Therefore, the apparent differences in Jenv-induced tumorigenesis in Akt1^−/−^, Akt2^−/−^, and Akt3^−/−^ mice are due to differences in the ability of Akt isoforms to transduce oncogenic signals.

One explanation for why Akt1 ablation significantly delays the induction of lung tumors in A_JE_JJenv infected mice is that Akt1 deficiency slows the growth of preneoplastic and early neoplastic lesions. Alternatively, Akt1 ablation may inhibit tumor initiation. The observation that Akt1 ablation leads to a decrease in cell proliferation and a marked increase in the number of apoptotic cells in A_JE_JJenv infected mice provides support for both hypotheses. Conversely, the dramatic increase in tumorigenesis induced by the ablation of Akt2 is due to both the enhancement of cell proliferation and inhibition of apoptosis caused by the loss of Akt2-transduced signals. These results suggest that inhibition of Akt1 protects against tumor initiation whereas Akt2 functions as a tumor suppressor in this model.

Our data show that both Akt1 and Akt2 are expressed in ATII cells, the putative tumor initiating cells, but that Akt1 is expressed at a higher level in these cells. It is possible that in the absence of Akt2, Akt1 no longer competes with Akt2 for proximity to membrane-located signaling complexes and thus becomes the dominant isoform responding to growth signals. Alternatively, Akt2 may keep Akt1 signaling in check by modulating the subcellular localization of Akt1 or by activating signaling pathways that modulate Akt1 mediated proliferative and survival signals. Akt2 might also act as a decoy substrate. Finally, we see a slight compensatory increase in Akt1 expression in the uninfected and tumor bearing Akt2^−/−^ and Akt3^−/−^ mice, which could contribute to the accelerated tumor development in these mice.

Delayed tumor onset and reduced tumor growth rate observed in the A_JE_JJenv infected Akt1^−/−^ mice was consistent with the findings of Hollander *et al* [19], which showed that loss of Akt1 prevented tumor initiation and tumor progression in both a carcinogen-induced and a mutant K-ras-induced mouse model of lung cancer. However, loss of Akt3 had a more dramatic impact on tumor multiplicity and size in the K-ras- and carcinogen-induced lung cancer models than it did in our model. The most notable difference between our results and those of Hollander et al. was the role of Akt2 as a putative tumor suppressor, such that deletion of Akt2 more than tripled lung tumor multiplicity and increased tumor initiation in our viral oncogene-induced mouse model of lung cancer. Therefore, in our model, Akt2, rather than Akt3, modulates the proliferative effects of Akt1 in lung tumorigenesis. One explanation for these differences is that Hollander et al. used mouse models of smoking induced lung cancer, whereas our viral oncogene-induced mouse model of lung cancer more closely resembles that of lung cancer of never smokers [7]. Major gender, molecular, and response to treatment differences in lung cancers arising in never smokers and smokers have recently been recognized, supporting the notion that they might in fact be different diseases [36]–[38]. Molecularly, these cancers differ as well. For example, K-ras mutations are more frequently found in adenocarcinomas arising in smokers while epidermal growth factor receptor mutations are more frequently found in adenocarcinomas arising in never smokers [39]–[44]. Of the few studies that have investigated the effect of Akt2 isoform ablation on tumor initiation and progression [19], [21], [45], only in mouse models of Neu- and PyMT-driven mammary carcinogenesis was Akt2 identified as a putative tumor suppressor [21]. Therefore, the activities of Akt isoforms appear to be highly tissue- and oncogene-dependent.

It is well known that Akt2 is critical for insulin signaling. Mice deficient in Akt2 develop hyperglycemia and hyperinsulinemia and are impaired in their ability to lower blood glucose in response to insulin [13]. Hyperinsulinemia is associated with increased incidence of neoplasia in several animal models of cancer [46]–[49]. In addition to insulin-mediated effects, hyperglycemia is thought to provide energy for malignant cell proliferation, which because of their dependence on aerobic glycolysis (Warburg effect) [50], favors cancer cell growth [51]. The use of thiazolidinediones to reduce hyperglycemia in type 2 diabetes has been associated with reduced risk of lung and other cancers [52], suggesting that removal of glucose as an energy substrate has a protective effect. While it is possible that loss of Akt2 might predispose mice to lung cancer independently of hyperactive insulin signaling, studies to understand the role of hyperglycemia in lung cancer progression are needed especially considering diabetes is a prevalent disease whose incidence is increasing globally [53].

The dramatic delay in lung tumor initiation observed in the Akt1^−/−^ mice could be due to impaired signaling through mTOR and GSK-3α/β leading to a reduction in cell growth and proliferation. In the case of the Akt3^−/−^ mice, only GSK-3α/β signaling was impaired which suggests that signals directing cell proliferation must be communicated through a different intermediate. Lastly, ablation of Akt2 does not increase mTOR activation as tumor burden increases. Rather, Akt2^−/−^ mice have a high level of basal mTOR activation, which might contribute to the exquisite susceptibility of these mice to lung tumorigenesis. Interestingly, unlike the Akt1^−/−^ and Akt3^−/−^ mice, in which GSK-3 activity is high and does not appear to be inhibited in response to Jenv signaling, Akt2^−/−^ mice possess a high level of basal GSK-3α/β phosphorylation which surprisingly decreases with increased tumor burden. It is well accepted that GSK-3 plays an important role in tumorigenesis and cancer progression. In contrast to many protein kinases, GSK-3 is active in resting cells and is inactivated when phosphorylated by Akt [54]. Thus, GSK-3 appears to function as a general repressor, phosphorylating its targets and keeping them turned off under resting conditions [55]. However, some studies suggest that GSK-3 could be a positive regulator of tumorigenesis. Ougolkov et al. demonstrated that inhibition of GSK-3 kinase activity using small molecule inhibitors or shRNA silencing leads to a decrease in pancreatic cancer cell proliferation and survival [56]. Similarly, Bilim et al. reported that genetic depletion or pharmacological inhibition of GSK-3 results in decreased renal cancer cell proliferation and survival [57]. Therefore, whether GSK-3 functions as a tumor suppressor or tumor promoter may depend on the tissue type in which the tumor originates.

Identification of Akt1 as the only Akt isoform required for both viral oncogene and mutant K-ras-mediated lung tumor initiation and progression legitimizes Akt1 as a possible therapeutic target for human NSCLC. Indeed, lung delivery of Akt1 shRNA in nanoparticles prevented nascent urethane-induced lung tumor formation by 35% with just a 30% decrease in Akt1expression [58]. This treatment also decreased both lung tumor number and tumor size in a K-ras^LA1^ transgenic mouse model [59].

In conclusion, the Akt pathway is a critical signaling node in cancer cell survival and proliferation and as such, is a target for chemotherapeutic intervention [60], [61]. The potential opposing roles of Akt1 and Akt2 in lung tumorigenesis suggest that development of non-selective Akt inhibitors may not be beneficial and in fact, may be detrimental, particularly in the case of lung cancer in never smokers. Our results could have important implications for how Akt inhibitors are used in the treatment of lung cancer.

## Materials and Methods

### Ethics statement

All work with animals was conducted in strict accordance with the Canadian Council of Animal Care (CCAC) guidelines. The animal use protocol was approved by the Animal Care Committee (ACC) of the University of Guelph. All efforts were made to minimize suffering.

### Mice

Akt1^−/+^ and Akt2^−/+^ mice [12], [13] were purchased from Jackson Laboratory (USA) and bred to obtain homozygous Akt1^−/−^ and Akt2^−/−^ knockout mice. Akt3^−/−^ mice [14] were generously provided by Dr. Morris Birnbaum (University of Pennsylvania). C57BL/6 mice were purchased from Charles River (Canada).

### AAV vectors

Construction of a recombinant AAV vector expressing the JSRV Env protein (A_JE_JJenv) has been described [7]. The packaging plasmid, pDGM6 [62], which encodes the AAV serotype 6 capsid, was kindly provided by Dr. David Russell (University of Washington). AAV vectors and packaging plasmids were propagated in Escherichia coli GT116 (InvivoGen). AAV vectors were produced by cotransfection of HEK 293 cells with vector and packaging plasmid as described previously [63]. AAV vector titers were determined by Southern blot [64].

### AAV vector administration to mouse lungs

Seven-week-old male mice were lightly anesthetized and given 1×10^11^ vector genomes (vg) of A_JE_JJenv in 2×50 ul doses via intranasal administration. Mice were euthanized at 12 (early neoplastic lesions), 20 (established tumors) and 32 (advanced neoplasms) weeks post vector administration (a minimum of 5 mice per time point). Lung, heart, liver, spleen, and kidney were harvested. Half the tissue was flash frozen in liquid nitrogen and the other half fixed in 2% paraformaldehyde and paraffin embedded.

### Immunohistochemical staining

Sections of paraffin embedded tissue were subjected to H&E and immunohistochemical staining as described previously [7]. Antibodies against SPC, CC10 and actin were purchased from Santa Cruz Biotechnology and the anti-Jenv monoclonal antibody was described previously [65]. Tissues were stained with anti-Ki67 antibody (Abcam) as described [66] and images were captured using a brightfield microscope at 200× magnification. Manual counts of Ki67 positive and negative nuclei were performed on three images per tissue section from a minimum of three mice per group.

### Western blot analysis

Monoclonal antibodies specific for Akt1, Akt2, Akt3, pan-Akt, phospho-Akt (Thr^308^), phospho-Akt (Ser^473^), phospho-GSK-3α/β (Ser^21/9^), p27 Kip1, phospho-PRAS40 (Thr^246^), phospho-TSC2 (Thr^1462^), phospho-mTOR (Ser^2448^), phospho-4E-BP1 (Ser^65^), phospho-MDM2 (Ser^166^), phospho-FoxO3a (Ser^318/321^), phospho-Bad (Ser^136^) and phospho-p44/42 MAPK (Thr^202^/Thr^204^) were purchased from Cell Signaling Technology. Lung tissue was homogenized in RIPA buffer (50 mM Tris pH 7.5, 150 mM NaCl, 1% Triton X-100, 0.1% SDS, 10 mM EDTA, 1% sodium deoxycholate) containing Na_3_VO_4_ (1 mmol/L), NaF (50 mM) and a cocktail of protease inhibitors (Sigma). Cell lysates were separated by gel electrophoresis (7–15% Tris-glycine gel) and transferred to PVDF membranes. Membranes were blocked in 5% skim milk-PBST and primary antibodies were incubated at a dilution of 1∶1000 in 1% BSA-PBST overnight at 4°C. Proteins were detected using HRP-conjugated secondary antibodies (Invitrogen) and Western Lightning Plus Chemiluminescence substrate (Perkin-Elmer). Images were captured using x-ray film.

### Terminal deoxynucleotidyl transferase–mediated dUTP nick end labeling (TUNEL) staining

TUNEL staining was performed on formalin-fixed, paraffin-embedded tissue sections using the In Situ Cell Death Detection Kit, POD (Roche Diagnostics) according to the manufacturer's instructions. For each slide, three images were captured at 200× magnification and TUNEL positive nuclei were counted manually and quantified as a percentage of the total number of nuclei present in each tissue image. A minimum of 3 slides per mouse per experimental group was assessed.

## Supporting Information

Figure S1 **Representative images of lung sections from WT, Akt1-/-, Akt2-/- and Akt3-/- C57BL/6 mice infected intranasally with 2.5×1010 vg of an AAV vector expressing the reporter gene, human placental alkaline phosphatase (hPLAP).** Mice were euthanized 4 weeks post-vector administration and lung tissue harvested, fixed and stained for alkaline phosphatase expression as described previously.** Yu DL, Linnerth-Petrik NM, Halbert CL, Walsh SR, Miller AD, Wootton SW (2011) JSRV and ENTV promoters drive gene expression in all airway epithelial cells of mice but only induce tumors in the alveolar region of the lung. J. Virol 85: 7535–7545.
(TIF)
