# Supplementary figures and images for: Opposing Functions of Akt Isoforms in Lung Tumor Initiation and Progression
Source: PLoS One. 2014 Apr 10;9(4):e94595. doi: 10.1371/journal.pone.0094595 (PMC3983215; doi:10.1371/journal.pone.0094595)

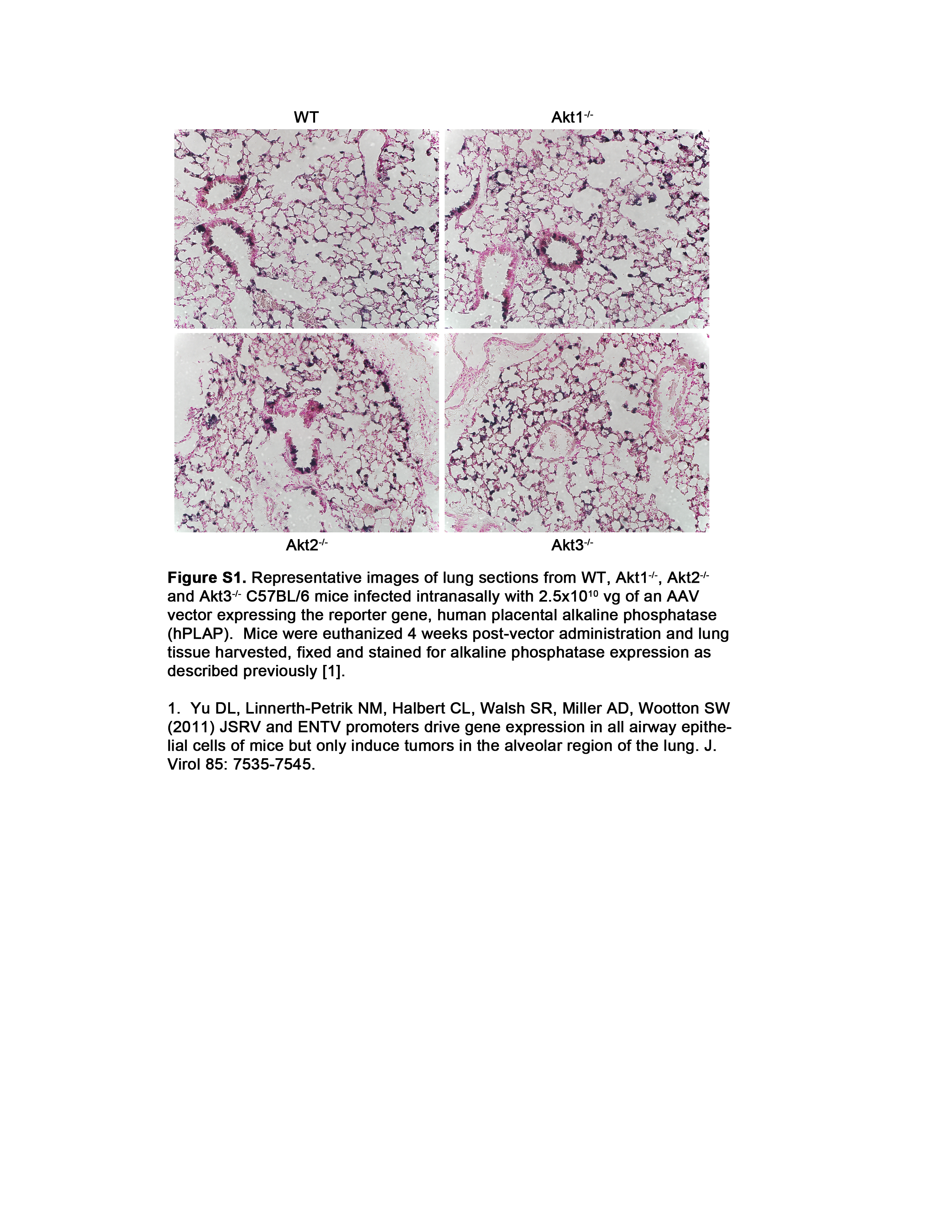

Supplement: Figure S1 — Representative images of lung sections from WT, Akt1-/-, Akt2-/- and Akt3-/- C57BL/6 mice infected intranasally with 2.5×10^10^ vg of an AAV vector expressing the reporter gene, human placental alkaline phosphatase (hPLAP). Mice were euthanized 4 weeks post-vector administration and lung tissue harvested, fixed and stained for alkaline phosphatase expression as described previously.** Yu DL, Linnerth-Petrik NM, Halbert CL, Walsh SR, Miller AD, Wootton SW (2011) JSRV and ENTV promoters drive gene expression in all airway epithelial cells of mice but only induce tumors in the alveolar region of the lung. J. Virol 85: 7535–7545. (TIF) [file pone.0094595.s001.tif]
